# Secreted Enzyme-Responsive System for Controlled Antifungal Agent Release

**DOI:** 10.3390/nano11051280

**Published:** 2021-05-13

**Authors:** Andrea Bernardos, Matěj Božik, Ana Montero, Édgar Pérez-Esteve, Esther García-Casado, Miloslav Lhotka, Adéla Fraňková, María Dolores Marcos, José Manuel Barat, Ramón Martínez-Máñez, Pavel Klouček

**Affiliations:** 1 Department of Food Science, Faculty of Agrobiology, Food and Natural Resources, Czech University of Life Sciences Prague, Kamýcká 129, 16500 Praha-Suchdol, Czech Republic; bozik@af.czu.cz (M.B.); anita_mmr@hotmail.com (A.M.); frankovaa@af.czu.cz (A.F.); 2 Instituto Interuniversitario de Investigación de Reconocimiento Molecular y Desarrollo Tecnológico (IDM), Universitat Politècnica de València, Universitat de València, Camino de Vera s/n, 46022 Valencia, Spain; mmarcos@qim.upv.es (M.D.M.); rmaez@qim.upv.es (R.M.-M.); 3 CIBER de Bioingeniería, Biomateriales y Nanomedicina (CIBER-BBN), Av. Monforte de Lemos 3–5, Pabellón 11, Planta 0, 28029 Madrid, Spain; 4 Department of Food Technology, Universitat Politècnica de València, Camino de Vera s/n, 46022 Valencia, Spain; edpees@upv.es (É.P.-E.); jmbarat@tal.upv.es (J.M.B.); 5 Department of Inorganic Technology, Faculty of Chemical Technology, University of Chemistry and Technology Prague, Technická 5, Praha 6, 16628 Prague, Czech Republic; esthergarciacasado@hotmail.es (E.G.-C.); miloslav.lhotka@vscht.cz (M.L.)

**Keywords:** nanoparticles, essential oil component, antimicrobial, antifungal, exogenous enzyme, *Aspergillus niger*

## Abstract

Essential oil components (EOCs) such as eugenol play a significant role in plant antimicrobial defense. Due to the volatility and general reactivity of these molecules, plants have evolved smart systems for their storage and release, which are key prerequisites for their efficient use. In this study, biomimetic systems for the controlled release of eugenol, inspired by natural plant defense mechanisms, were prepared and their antifungal activity is described. Delivery and antifungal studies of mesoporous silica nanoparticles (MSN) loaded with eugenol and capped with different saccharide gates—starch, maltodextrin, maltose and glucose—against fungus *Aspergillus niger*—were performed. The maltodextrin- and maltose-capped systems show very low eugenol release in the absence of the fungus *Aspergillus niger* but high cargo delivery in its presence. The anchored saccharides are degraded by exogenous enzymes, resulting in eugenol release and efficient inhibition of fungal growth.

## 1. Introduction

Botanical systems have developed the intriguing ability to respond to various stimuli due to long-term survival competition [1]. Volatile compounds produced by plants are one of the simplest but most effective and ubiquitous constituents of plant defense systems. Thanks to these compounds, many plants possess insecticidal, antifungal, antibacterial, acaricidal and cytotoxic activities [2,3,4] and are intentionally used by animals to protect themselves, as documented in the case of elephants, pigs and apes [5]. However, the utilization of plants as bioactive agents was mastered by humans. Plant volatiles have been extracted in the form of essential oils and used as antimicrobial agents in health care, food production, or cosmetics for thousands of years [6]. However, extraction compromises one of the most important determinants of the effectiveness of plant volatiles, i.e., their release. Plants produce these compounds in advance and store them until needed, either in special organs, such as glandular trichomes and essential oil ducts, or in non-active chemical forms, such as glycosides. In either case, the trigger for release is similar: enzymatic activity. Stored compounds are released when the storage tissues of the plant are damaged by the enzymes of the attacking microorganism. In the case of glycosides, such as those found in garlic or mustard, cell damage induces internal enzymes that cleave the glycosidic bond and release the active form of the molecule to target the attacker [7]. To mimic this natural behavior, different encapsulation technologies have been proposed. However, these technologies are usually only “slow release”, which is not really “controlled”. We propose that the precise control of the delivery of such volatile and reactive molecules, inspired by the delivery mechanisms found in plants [8], is crucial for their effective utilization in various applications, such as novel food preservatives or sustainable plant protection agents [9,10].

The development of new formulations to control the delivery of volatile and reactive molecules has given rise to new concepts related to the design of micro or nanodevices that allow the targeted delivery of bioactive compounds [11,12,13]. Traditional delivery systems are usually based on organic polymers, such as β-cyclodextrins, that release their cargo via degradation of the polymeric matrix or by controlled-release diffusion processes [14,15]. Recently, much attention has been given to mesoporous silica nanoparticles (MSNs) as inorganic scaffolds for the loading and delivery of drugs and other bioactive molecules [16,17,18,19]. The pore and particle sizes of MSNs can be tailored for different applications to provide unique features such as stability, biocompatibility, and large load capacity for on-command delivery applications. The functionalization of MSNs with “molecular gates” is a particularly promising research field [20]. Capping the pores of MSNs with molecular gates that can be opened with different stimuli would guarantee the complete delivery of the cargo at the action point [21]. Different external stimuli (ionic, photo-chemical, electrochemical, changes in the polarity, presence of biomolecules) [22] have been employed for the controlled release of a variety of guest molecules (cytotoxic agents, proteins, DNA and RNA fragments, enzymes) [23,24,25,26]. Controlled release of a substance only in the presence of a specific stimulus is thus an emerging and innovative idea [27]. Biomimetics is an interdisciplinary field in which principles from chemistry and biology are applied to the synthesis of materials or synthetic systems whose functions mimic biological processes [28]. In plant–pathogen interactions, the enzymes triggering volatile compound release can be of fungal, bacterial or plant origin from the family of glycoside hydrolases, such as amylase [29,30,31]. These enzymes, produced by fungi including *A. niger*, can degrade the cell wall of the plant storage organs filled with the essential oils and release active compounds to inhibit or even kill the attacking microorganism. The use of specific saccharides as molecular gates would mimic this natural mechanism of plant defense and would have great potential for applications in the food, agricultural, cosmetic and pharmaceutical industries by allowing the encapsulation of essential oil components (EOCs) as natural antimicrobials for precisely controlled delivery [32].

Accordingly, the present work describes the synthesis of MSNs loaded with eugenol, an EOC, and functionalized with different ‘saccharide gates’ covalently bonded to block the pores. In the presence of microorganisms, exogenous amylases hydrolyze the saccharide, releasing the EOC. This study aimed to assess whether nanoscopic mesoporous silica particles functionalized with different saccharide derivatives on the pore outlets can be used as carriers for EOCs to control their release and volatility via an external trigger in the form of a fungal enzyme.

## 2. Materials and Methods

### 2.1. Chemicals and Biological Materials

Tetraethyl orthosilicate (TEOS), *n*-cetyltrimethylammonium bromide (CTABr), sodium hydroxide (NaOH), eugenol 99%, 3-aminopropyltriethoxysilane, ethanol anhydrous, deuterium oxide (D_2_O), glucose, maltose, maltodextrin 18% and starch were purchased from Sigma-Aldrich (Prague, Czech Republic). *Aspergillus niger* (*A. niger*) ATCC 6275 was purchased from the Czech Collection of Microorganisms (Faculty of Science, Masaryk University, Brno, Czech Republic). Sabouraud Dextrose Agar (SDA) and Muller-Hinton broth (MHB) for *A. niger* were purchased from Oxoid (Brno, Czech Republic). All chemicals and biological materials were used as received.

### 2.2. Synthesis of Nanoparticles

The MCM-41-type mesoporous silica nanoparticles were synthesized using the following procedure: n-cetyltrimethylammonium bromide (CTABr, 2.00 g, 5.48 mmol) was first dissolved in 960 mL of deionized water. NaOH (aq) (2.00 M, 7.00 mL) was added to the CTABr solution, and the temperature of the solution was adjusted to 95 °C. TEOS (10.00 mL, 44.78 mmol) was then added dropwise to the CTAB solution. The mixture was stirred for 3 h to give a white precipitate, and the solid product was collected by centrifugation at 9500 rpm and washed with ethanol and deionized water. The final solid was dried at 60 °C (as-made solid). Finally, to prepare the final porous material (MSN), the as-made solid was calcined at 550 °C under an oxidant atmosphere for 5 h to remove the surfactant.

### 2.3. Eugenol Encapsulation in the Silica Nanoparticles

Silica loading with eugenol was achieved via vapor adsorption by mixing 100 mg of eugenol with the 100 mg culminated MSN in a tightly closed vial. The mixture was incubated in an oven at 40 °C for 24 h with continuous shaking. The amount of eugenol loaded in nanoparticles was determined gravimetrically and by GC-FID after alkaline hydrolysis and hexane extraction (details see below).

### 2.4. Molecular Gates Synthesis

3-Aminopropyltriethoxysilane (5.85 mL, 25 mmol) was solved in ethanol and added to a suspension of saccharide (5.4 g) in ethanol (final volume 250 mL) to create a covalent bond between the anomeric C-OH from the sugar and the NH_2_ group from the alkoxysilane molecule [24]. The mixture was stirred at room temperature for 24 h and heated at 60 °C (30 min). The solvent was evaporated under reduced pressure giving a white solid. ^1^H-NMR (300 MHz, D2O): δ 0.42 (t, 2 H, –CH_2_–Si–), 1.02 (t, 9 H, CH_3_–CH_2_–O–Si–), 1.53 (m, 2 H, –CH_2_–CH_2_–Si–), 2.74 (t, 2 H, –NH–CH_2_–CH_2_–CH_2_–Si–), 3.20-3.77 (m, n H, starch hydrolyzed, CH_3_–CH_2_–O–Si–), 5.13 (d, 1 H, –O–CH–O–) ppm [32].

### 2.5. Functionalization on the Silica Support

In a typical synthesis, 200 mg of MSN-Eu was suspended in 25 mL of water in a round-bottomed flask under an inert atmosphere. Then, an excess of the corresponding saccharide derivative (200 mg) was added, and the final mixture was stirred for 5.5 h (at room temperature). Finally, the Saccharide-MSN-Eu solid was collected by filtration, washed with water, and then dried for 12 h.

### 2.6. Methods for Nanoparticle Characterization

Powder X-ray diffraction (PXRD), N_2_ adsorption–desorption, dynamic light scattering (DLS), zeta potential measurements, transmission electron microscopy (TEM), field emission scanning electron microscopy (FESEM), thermogravimetric analysis (TGA), elemental analysis (EA) and nuclear magnetic resonance (NMR) were employed to characterize the materials. X-ray measurements were performed on a Bruker AXS D8 Advance diffractometer using Cu-Kα radiation (Bruker, Coventry, UK). The adsorption isotherms were fit to obtain the BET (Brunauer–Emmett–Teller) surface area [33], the micropore volume was derived using the t-plot method, and the pore size distribution was obtained with the BJH (Barrett–Joyner–Halenda) method [34]. Samples were stored at 60 °C under vacuum for 33 h before adsorption measurements.

DLS studies and zeta potential (ζ) analysis were conducted at 25 °C using a Malvern Zetasizer Nano ZS (Malvern Instruments, Malvern, UK). Samples were suspended in distilled water at a concentration of 1 mg mL^−1^. Before each, the samples were sonicated for 2 min to prevent aggregation before starting the measurement. The zeta potential was calculated from the particle mobility values by applying the Smoluchowski model. The average of five recordings was described as the zeta potential. TEM images were obtained with a 100 kV Philips CM10 microscope (Amsterdam, The Netherlands). Equilibrium adsorption isotherms of nitrogen were measured at 77 K using static volumetric adsorption systems (ASAP 2020 analyzer, Micrometrics, Norcross, GA, USA). FESEM images were acquired with a Zeiss Ultra 55 (Carl Zeiss NTS GmbH, Oberkochen, Germany) and observed in secondary electron mode. TGA analysis was carried out on a TGA/SDTA 851e Mettler Toledo balance using an oxidant atmosphere (air, 80 mL min^−1^) with a heating program. This program consists of a heating ramp of 10 °C per minute from 273 to 373 K followed by an isothermal heating step at this temperature for 60 min in a nitrogen atmosphere (80 mL min^−1^). At that time, the program was allowed to continue with a dynamic heating segment from 373 to 1273 K in an oxidant atmosphere (air, 80 mL min^−1^) and with an isothermal heating step for 30 min. Elemental analysis was performed in a CE Instrument EA-1110 CHN Elemental Analyzer (Wigan, UK). For quantification of encapsulated eugenol by GC-FID, 1 mg of material was dissolved in 100 µL of 10 M NaOH, which degraded the silica structure of MSNs, and the solution was then neutralized by adding 105 µL of 35% HCl. The content was extracted twice with 500 µL of hexane and analyzed by GC-FID (6890 Network GC system, Agilent Technologies, Palo Alto, CA, USA) with external calibration (*R*^2^ = 0.9998, recovery 93%). The inlet and detector temperatures were 250 °C, and 1 µL of an extract was injected in splitless mode onto a HP-5MS column (5% phenyl methyl siloxane, 30 m × 250 μm × 0.25 μm). The oven program began at 100 °C for 1 min and then ramped up from 20 °C/min to 200 °C for 4 min. ^1^H-NMR spectra were acquired with Avance I™ 400 MHz equipment (Bruker, Coventry, UK). The ^1^H-NMR studies were carried out in deuterium oxide.

### 2.7. Preparation of the A. niger Inoculum

A spore suspension was prepared by washing an *A. niger* culture with 2 mL of sterile MHB (Mueller-Hinton Broth, Oxoid, Brno, Czech Republic) containing 1% Tween 80 surfactant (Carl Roth, Karlsruhe, Germany). The inoculation solution was standardized to a density value of 0.5 McFarland (densitometer McFarland type DEN-1B, Biosan, Rīga, Latvia), which corresponds to 5 × 10^6^ CFU mL^−1^, and diluted to a final concentration of 5 × 10^4^ CFU mL^−1^ [35].

### 2.8. Delivery Study

Delivery tests were performed in 20-mL headspace (HS) vials closed with a crimp cap PTFE/silicon septa (Supelco, Bellefonte, PA, USA). Each test included two vials to study eugenol release in the absence and presence of *A. niger*. The first vial was prepared with 2 mL of SDA (Sabouraud Dextrose Agar, Oxoid, Brno, Czech Republic) media. Next, 5 mg of Maltodextrin-MSN-Eu was vigorously mixed with 100 µL of water at pH 7.5 in an Eppendorf tube and evenly dispersed onto the agar. The second vial was prepared in the same way, but 50 µL of the *A. niger* inoculum was also added. The vials were closed and incubated in a dark box at 25 °C, and eugenol release was monitored by SPME-GC-FID analysis on days 1, 4, 8, and 15. For headspace analysis, a solid phase microextraction (SPME) manual holder with 100 μm PDMS (polydimethylsiloxane) fiber (Supelco, Bellefonte, PA, USA) was used. Gas chromatography coupled with flame ionization detection (GC-FID) was used to analyze cargo release. Analyses were carried out using an Agilent Technologies (Agilent Technologies, Palo Alto, CA, USA) 6890 Network GC system with an RTX-5 column (20 m × 0.18 mm × 0.20 μm) and an injector temperature of 250 °C in splitless mode. The oven temperature started at 50 °C and was increased in two ramps: the first at 10 °C/min to 160 °C and the second at 30 °C/min to a maximum of 250 °C. The detector temperature was 250 °C. The experiment was performed in triplicate.

### 2.9. Minimum Inhibitory Dose Assay

The minimum inhibitory dose (MID) test was performed in vitro in Petri dishes with a diameter of 60 mm by the modified disc diffusion method [36]. Into each dish, 5 mL of SDA was poured. Then, 50 µL of the inoculum was vigorously and thoroughly mixed with the encapsulated and pure substances in an Eppendorf tube containing 100 µL of sterile PBS at pH 7.5 and evenly dispersed onto the agar. The antifungal activities of doses of 0.5 mg, 1 mg, 1.5 mg and 2 mg of eugenol in pure form were compared with those of the encapsulated materials, that is, 1 mg, 2 mg, 3 mg and 4 mg of MSN-Eu and 5 mg, 10 mg, 15 mg and 20 mg of Saccharide-MSN-Eu. The exact content of eugenol in the different saccharide-gated materials is shown in Table 1. Inoculated Petri dishes with or without empty MSN were used as controls. The Petri dishes were closed but not sealed, allowing vapors to escape. All Petri dishes were incubated in a dark box at 25 °C with one open Petri dish containing water to maintain high humidity. The MID was the lowest dose with a complete lack of fungal growth and was evaluated on days 3, 6 and 15. All formulations were tested in triplicate.

### 2.10. Antimicrobial Assay Visualization

The antimicrobial activity of 1 mg of the substance in pure form was compared to those of the encapsulated materials, i.e., 2 mg of MSN-Eu and 10 mg of Saccharide-MSN-Eu, as described for the MID tests above. Inoculated Petri dishes with or without empty nanoparticles were used as controls. All Petri dishes were incubated in a dark box at 25 °C with high humidity. To visualize *A. niger* growth inhibition, the Petri dishes were documented by taking photographs with a digital camera (Cannon EOS 350D, Tokyo, Japan) under standard light conditions (4000 K) with a white background on days 0, 1, 4, 8, 11 and 15. The luminosity of each Petri dish picture was analyzed as a function of time. The value of relative luminosity was calculated by dividing the luminosity of each sample by the luminosity of the growth control (without any EOCs) on that particular day. The Zoner Photo Studio software was used to analyze all pictures taken during the experiment (version 15, Zoner Software a.s., Brno, Czech Republic). The evaluation was carried out in triplicate, and the standard deviation was calculated for each sample.

## 3. Results and Discussion

### 3.1. Gated Materials

In this study, a biomimetic system for controlled release of natural antifungal agent eugenol was designed. As it was described above, biomimetics is an interdisciplinary field in which principles from chemistry and biology are united to develop synthetic systems acting as mimic biological processes. Biomimetic designs were primary used in regenerative medicine, tissue engineering and drug delivery; nowadays, they are increasingly used in agriculture for pest control [37,38] and food preservation [39,40]. The aim of this study was to design biocompatible, easy-to-prepare and low-cost molecular-gated capping systems to synthesize simple gated systems to be used efficiently in controlled release applications.

**Figure 1:**
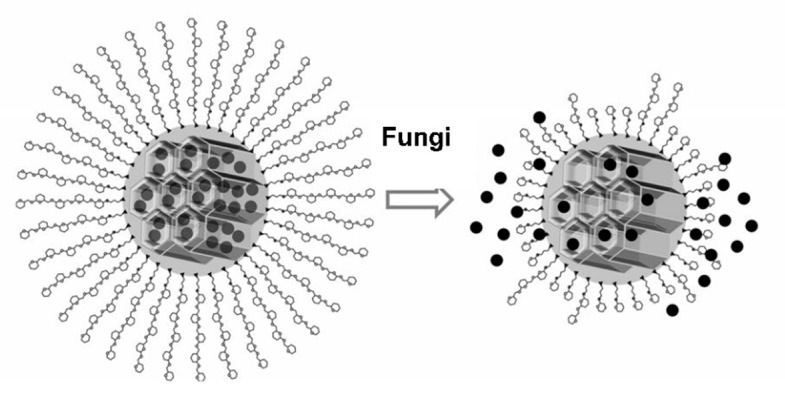
Representation of the Saccharide-MSN-Eu system in the presence of exogenous fungal enzymes.

The delivery system presented in this work is based on MSN functionalized with saccharide “molecular gates” and loaded with the EOC eugenol. In detail, the gate-like functional hybrid systems presented in this work consist of MCM-41 type-based nanoparticles functionalized with different “saccharide” derivatives on the pore outlets and containing the EOC eugenol (Eu) in the mesopores. Microorganisms such as the fungus *A. niger* produce the exogenous amylases required for saccharide hydrolysis [41] and allow the release of the EOC (see Figure 1).

An MCM-41 type inorganic scaffold in nanoparticle form containing mesopores in the 2–3 nm range was used (vide infra). Eugenol was loaded into the MCM-41 type pores as the agent for antimicrobial tests and delivery to evaluate the effectiveness of the gated systems. Among the different “molecular gate” cap possibilities, the focus was on the use of saccharides. In order to avoid the complex synthetic routes to saccharide derivatives, glucose, maltose, maltodextrin 18% and starch, commercially available products, were used. Saccharides and their mixtures are readily available simple carbohydrate polymers that have been widely used as food components and biodegradable systems for the stabilization of certain species [42]. Each of these saccharides presents different values of dextrose equivalent percentage: starch (close to 0% dextrose equivalent), maltodextrin (18% dextrose equivalent), maltose (50% dextrose equivalent), and glucose (100% dextrose equivalent). Anchoring of these saccharide derivatives on the external surface of the MSNs loaded with eugenol resulted in the preparation of the following systems: Glucose-MSN-Eu, Maltose-MSN-Eu, Maltodextrin-MSN-Eu and Starch-MSN-Eu.

The anchoring of the saccharide derivatives was expected to inhibit cargo release due to the formation around the pore outlets of a thick hydrogen-bonding, interaction-based “saccharide” system [24]. These saccharide gates were created to avoid fast evaporation and allow precise timing of eugenol delivery. We hypothesized that in the presence of exogenous enzymes from fungi (containing amylases able to hydrolyze the 1→4 glycosidic bonds between β-D-glucoses present in starch, maltodextrin and maltose), hydrolysis of the ‘saccharide’ gates would result in uncapping of the pores and delivery of the entrapped molecule (vide infra).

### 3.2. Characterization

**Figure 2:**
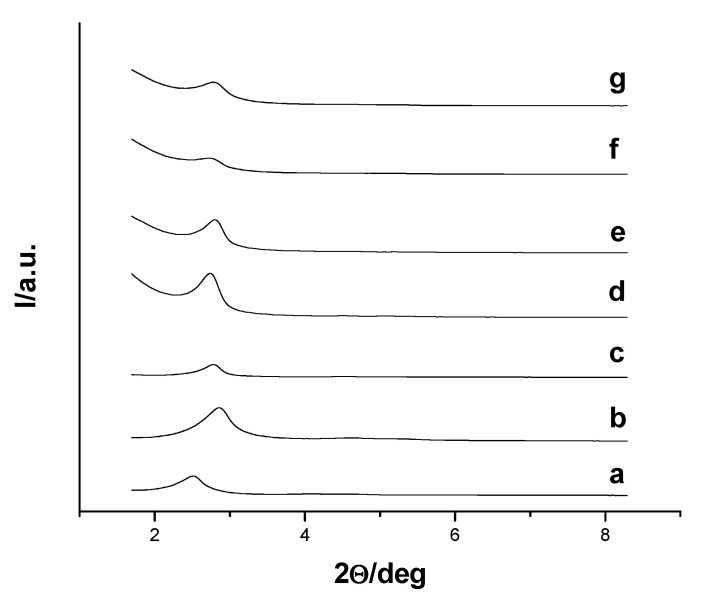
Powder X-ray diffraction patterns of the scaffolds and systems: (**a**) MCM-41 type as-made, (**b**) MSN, (**c**) MSN-Eu, (**d**) Glucose-MSN-Eu, (**e**) Maltose-MSN-Eu, (**f**) Maltodextrin-MSN-Eu and (**g**) Starch-MSN-Eu.

The prepared systems were characterized using standard techniques. The powder X-ray diffraction (PXRD) patterns of the nanoparticles MCM-41 type as-made, MSN, Glucose-MSN-Eu, Maltose-MSN-Eu, Maltodextrin-MSN-Eu and Starch-MSN-Eu are presented in Figure 2. PXRD of MCM-41 type as-made (Figure 2, curve a) revealed four low-angle reflections typical of a hexagonal ordered array that can be indexed as (100), (110), (200) and (210) Bragg peaks. The condensation of silanol groups during the calcination step is clearly reflected in a significant shift of the (100) peak to higher 2θ values (Figure 1, curve b). The presence of the (100) reflection peak in the PXRD patterns of the saccharide-capped systems demonstrated that both loading and functionalization processes did not alter the mesoporous structure of the scaffold (curves c–g in Figure 2).

**Figure 3:**
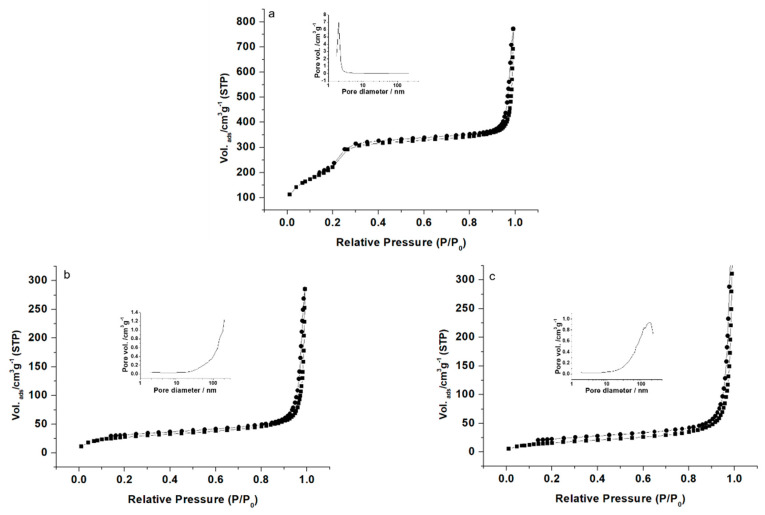
Nitrogen adsorption–desorption isotherms for (**a**) MSN, (**b**) MSN-Eu and (**c**) Maltodextrin-MSN-Eu. The insets in each graph show the pore size distributions of the systems.

The N_2_ adsorption–desorption isotherms of the nanoparticulate MCM-41 type calcined material are shown in Figure 3a. The curves for these mesoporous scaffolds are typical of an adsorption step at intermediate P/P0 value (0.1–0.3) and correspond to type IV isotherms, in which the observed step involves nitrogen condensation inside the mesopores. The non-appearance of a hysteresis loop in this interval and the narrow BJH pore distribution suggest the existence of constant cylindrical mesopores (pore diameter of 2.4 nm and pore volume of 1.1 cm^3^ g^−1^ calculated by using the BJH model on the adsorption branch of the isotherm). Application of the BET model resulted in a total specific surface value of 825 m^2^ g^−1^. In addition to this adsorption step associated with the micelle-generated mesopores, a second feature appears in the isotherm at high relative pressure (P/P0 > 0.8). This adsorption corresponds to filling of the large voids among the particles and, therefore, textural-like porosity. In this situation, the curves show a characteristic H1 hysteresis loop and a wide pore size distribution. The N_2_ adsorption–desorption isotherms of MSN-Eu and Maltodextrin-MSN-Eu are typical of mesoporous systems, in which the mesopores are nearly completely filled (Figure 3b,c). Consequently, relatively low N_2_ adsorbed volume and surface area (Table 2) values were calculated. In fact, the curves for these systems are flat when compared with those of the MCM-41 type parent material (at the same scale), indicating significant pore blocking and consequently, a lack of appreciable mesoporosity.

**Figure 4:**
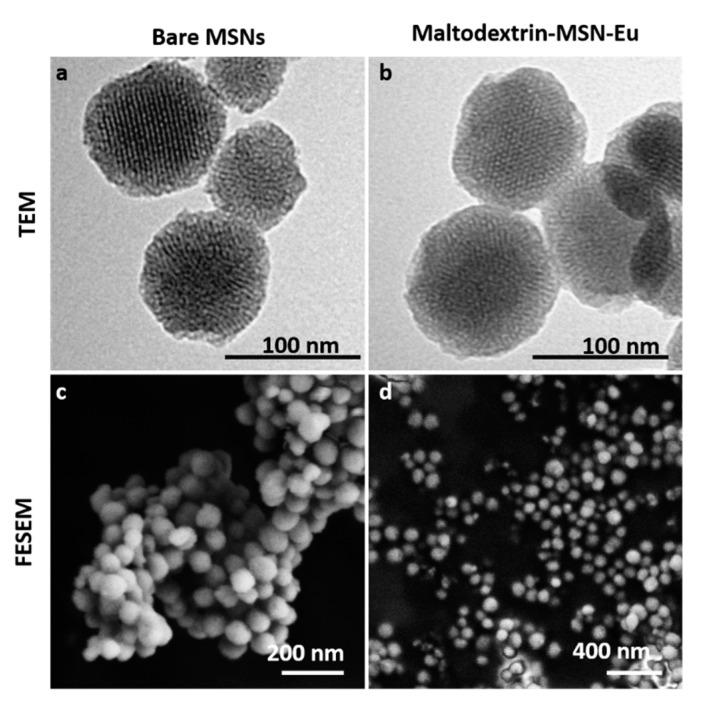
TEM images of bare MSN (**a**) and Maltodextrin-MSN-Eu (**b**), and FESEM images of bare MSN (**c**) and Maltodextrin-MSN-Eu (**d**), showing the typical shape and structured pores of the MCM-41 matrix.

DLS studies of MCM-41 type, MSN-Eu and Maltodextrin-MSN-Eu were led at 25 °C, with a previous sonicated suspension of the nanomaterials in PBS (phosphate-buffered saline, Sigma-Aldrich) at a concentration of 0.01 mg mL^−1^. The results are listed in Table 3. Zeta potential measurements of the suspended materials were also carried out (Table 3). Bare MCM-41 type exhibited a negative zeta potential value that changed to positive after functionalization with maltodextrin. This positive charge is likely important for antimicrobial activity; since the fungal cell wall is negatively charged, strongly cationic nanoparticles (more than +30 mV) will have greater membrane permeation. The mesoporous structure of the final functionalized systems was also confirmed by TEM and FESEM analysis, in which the typical channels of the MCM-41 matrix were visualized as alternating black and white stripes (Figure 4). The FESEM images showed that the prepared MCM-41-based materials were spherical particles with diameters of approximately 100 nm (Figure 4).

Moreover, the organic content of eugenol and saccharides in the different saccharide-gated materials is shown in Table 1.

### 3.3. Fungal Enzyme-Responsive Controlled Release

Several experiments were carried out to study enzyme-responsive controlled release by the saccharide-capped systems. The capability of Starch-MSN-Eu, Maltodextrin-MSN-Eu, Maltose-MSN-Eu and Glucose-MSN-Eu to control the release of eugenol from the capped systems in the presence of enzymes from the fungus *A. niger*, as depicted in Figure 1, was analyzed.

**Figure 5:**
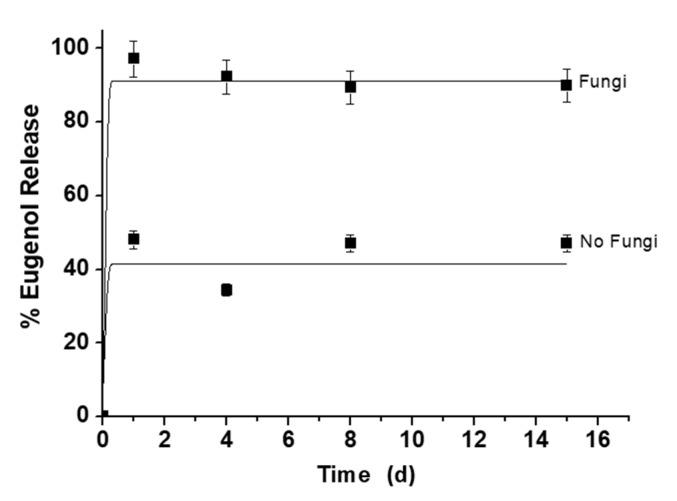
Kinetic release profile of eugenol from Maltodextrin-MSN-Eu in the absence and presence of fungi in the SPME assay after 15 d.

First, a model delivery study with the solid Maltodextrin-MSN-Eu was carried out to investigate the gating properties of the saccharides in the absence and presence of fungi. In a typical experiment, 10 mg of Maltodextrin-MSN-Eu were dispersed in vials containing agar in the presence and absence of *A. niger*. The vials were sealed to prevent vapor release and incubated in a dark box at 25 °C for 15 d to demonstrate the long-term effect of the designed MSN. At given times, vapor fractions were taken from each sample. The eugenol content in each aliquot was measured by SPME-GC-FID, as shown in Figure 5. Maltodextrin-MSN-Eu showed low release of eugenol (40%) in the absence of fungi. By contrast, in the presence of fungi, Maltodextrin-MSN-Eu delivered 100% of its cargo presumably due to uncapping of the pores through selective hydrolysis of the 1→4 glycosidic bond in the saccharide chains by exogenous fungal amylases. Both release curves (Maltodextrin-MSN-Eu with and without fungi) show a rapid release in the first two days, which is probably caused by the large amount of eugenol that is on the surface of the particles.

Moreover, minimum inhibitory dose (MID) tests were carried out with the set of MSNs capped with the different saccharides as molecular gates over 15 d. The results for the capped MSNs are compared with those for the eugenol-free control, pure eugenol and MSN-Eu (eugenol encapsulated in MSN without saccharide gate) and are shown in Table 4 and Figure S1 (Supplementary Materials). The antifungal activities of doses of 0.5, 1.0, 1.5, 2.0 and 2.5 (corresponding to (i) 0.5 mg, 1 mg, 1.5 mg, 2 mg and 2.5 mg of eugenol in pure form; (ii) 1 mg, 2 mg, 3 mg, 4 mg and 5 mg of MSN-Eu; and (iii) 5 mg, 10 mg, 15 mg, 20 mg and 25 mg of Saccharide-MSN-Eu) were evaluated. As shown in Table 4, the systems based on MSN showed better antifungal effects than pure eugenol when assayed after 3 d; after 6 d, antifungal activity was confirmed for Maltose-MSN-Eu and Maltodextrin-MSN-Eu and at higher doses of MSN-Eu; and after 15 d, antifungal activity was confirmed for only two of the six tested systems (Maltose-MSN-Eu and Maltodextrin-MSN-Eu).

The contents of eugenol in all of the systems determined by GC-FID are shown in Table 1. Taking this information into account, after 15 d, the MIDs of encapsulated Maltose-MSN-Eu and Maltodextrin-MSN-Eu, the most active systems, were 10 mg (approximately 0.76 mg of eugenol) and 5 mg (approximately 0.41 mg of eugenol), respectively. At the same time point, the other materials were not active even at the highest doses of 2.5 mg of pure eugenol; 5 mg of MSN-Eu (2.5 mg of active eugenol); 25 mg Glucose-MSN-Eu (approximately 1.725 mg of eugenol); and 25 mg of Starch-MSN-Eu (approximately 0.9 mg of eugenol). The eugenol was probably volatilized in the case of MSN-Eu and Glucose-MSN-Eu due to insufficient blockage of the pores. On the contrary, in the case of Starch-MSN-Eu, the fungi were probably not able to hydrolyze the high number of *o*-glycosidic bonds compared with the limited number of glycosidic bonds in Maltose-MSN-Eu and Maltodextrin-MSN-Eu.

**Figure 6:**
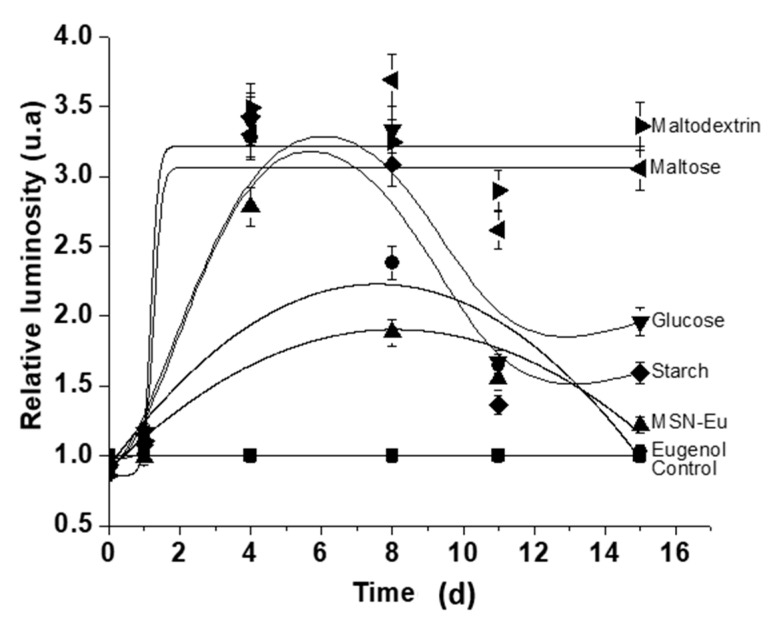
The degree of antifungal activity according to luminosity measurement for 15 d. The values are the ratios of the respective material to the growth control. The higher the relative luminosity, the greater the difference in inhibition compared with the growth control.

Furthermore, the antimicrobial activities of 1 mg of the pure eugenol, 2 mg of MSN-Eu and 10 mg of each Saccharide-MSN-Eu were digitally monitored for 15 d and compared with a control. At day 15, there was a complete antifungal effect in the presence of Maltose-MSN-Eu and Maltodextrin-MSN-Eu, whereas the remaining formulations maintained very low antifungal activity (Figure 6). Whereas pure eugenol, eugenol encapsulated in non-gated MSN, and eugenol encapsulated in MSNs gated with glucose or starch partially prevented *A. niger* growth during the first 8–10 d only, particles gated with maltose and maltodextrin displayed complete inhibition during the whole duration of the test. These results are in agreement with those of MID tests. While bare MSN, pure eugenol, eugenol encapsulated in non-gated MSN, and eugenol encapsulated in MSN gated with glucose or starch did not prevent *A. niger* growth, particles consisting of eugenol encapsulated in MSN gated with maltose and maltodextrin did. The antimicrobial activities of eugenol are widely accepted [43] and its antifungal mechanism of action was recently proposed [44]. Additionally, experiments with its encapsulation and/or nanoparticles preparation are reported [45,46,47], mainly against food-borne or human pathogens, showing its wide antimicrobial potential.

In this study, Maltodextrin-MSN-Eu showed better antifungal effect compared with pure eugenol, MSN-Eu, Glucose-MSN-Eu, and Starch-MSN-Eu. Both materials were able to maintain their antifungal activity against *A. niger* for 15 d. Eugenol was demonstrably more effective in a state of encapsulation and capped with maltose and maltodextrin gates, than when in the pure state, in encapsulated form or when the sugar gate is glucose (one monosaccharide) or when starch (polysaccharide formed by n molecules of glucoses). The well-defined amount of glucoses in maltose and maltodextrin appears to be the reason for the good effectivity of them against *A. niger*.

These results indicate that nanoparticles used for controlled release of essential oil components are suitable materials for the delivery of different cargoes, such as biocides, pesticides or antimicrobial agents, and applied into agricultural and food industries. Moreover, the possibility of using secreted-enzymes from microbials opens a wide range of new perspectives and possibilities in the development of biocompatible delivery systems using silica mesoporous supports applied in the agri-food field.

## 4. Conclusions

In this study, a new bioinspired release mechanism for eugenol, a representative antifungal EOC, was developed in which release was triggered by enzymes expressed by the target itself, the fungus *A. niger*. After encapsulation in a porous silica scaffold capped with a maltose or maltodextrin gate, the antifungal activity of eugenol against *A. niger* was maintained until the end of the experiment, i.e., 15 d. Shorter or longer saccharide chains were not effective. The systems showed eugenol release in the presence of fungi due to hydrolysis of the ‘saccharide gate’ by the exogenous fungal amylases. The use of exogenous enzymes from fungi as a specific stimulus in the controlled release of bioactive substances from mesoporous silica nanoparticles capped with ‘saccharides’ has not been reported previously. This approach is one of the first to mimic biological systems for controlled release of a natural compound.

## Figures and Tables

**Table 1:** Content (α) of saccharide gates and eugenol per gram of MSNs.

System	α_saccharide_ (g/g MSN)	α_eugenol_ (g/g MSN)
MSN-Eu	-	0.5
Glucose-MSN-Eu	0.195	0.069
Maltose-MSN-Eu	0.188	0.076
Maltodextrin-MSN-Eu	0.175	0.082
Starch-MSN-Eu	0.294	0.036

**Table 2:** BET-specific surface values, pore volumes and pore sizes calculated from the N_2_ adsorption–desorption isotherms for selected materials.

	*S*_BET_ (m^2^ g^−1^)	Pore Volume *^a^* (cm^3^ g^−1^)	Pore Size *^a,b^* (nm)
MSN (MCM-41 type)	825	1.1	2.4
MSN-Eu	101.5	0.41	-
Maltodextrin-MSN-Eu	67.7	0.59	-

*^a^* Pore volumes and pore sizes associated with intraparticle mesopores. *^b^* Pore size estimated by using the BJH model applied on the adsorption branch of the isotherm.

**Table 3:** Hydrodynamic diameter of materials measured by DLS and zeta potential measurements.

	Hydrodynamic Particle Diameter (nm)	Zeta Potential (mV)
MSN (MCM-41 type)	105 ± 2	−39.5 ± 0.9
MSN-Eu	108 ± 3	−40.0 ± 0.9
Maltodextrin-MSN-Eu	174 ± 8	42 ± 2

**Table 4:** Minimal inhibitory doses (MIDs, mg/Petri dish) of eugenol and mesoporous silica systems with different saccharide gates.

	3 d	6 d	15 d
Eugenol	2.5	N	N
MSN-Eu	0.5	1.5	N
Glucose-MSN-Eu	0.69	N	N
Maltose-MSN-Eu	0.38	0.76	0.76
Maltodextrin-MSN-Eu	0.41	0.41	0.41
Starch-MSN-Eu	0.54	N	N
N: no fungal inhibition	-	-	-

## Data Availability

Not applicable.

## Supplementary Materials

The following are available online at https://www.mdpi.com/article/10.3390/nano11051280/s1, Figure S1: Picture of antifungal activity of control (a), pure eugenol (b), eugenol encapsulated in silica SMPS-Eu (c), eugenol encapsulated in silica and capped with sugar derivative: Glucose-SMPS-Eu (d), Maltose-SMPS-Eu (e), Maltodextrin-SMPS-Eu (f) and Starch-SMPS-Eu (g) after 15 days.
