# Supplementary material for: Secreted Enzyme-Responsive System for Controlled Antifungal Agent Release
Source: Nanomaterials (Basel). 2021 May 13;11(5):1280. doi: 10.3390/nano11051280 (PMC8153022; doi:10.3390/nano11051280)
Supplement: Supplementary file 1 [file nanomaterials-11-01280-s001.zip › nanomaterials-1194864-supplementary.pdf]

nanomaterials

Supplementary Materials for

Secreted Enzyme-Responsive System for Controlled Antifungal Agent Release

Andrea Bernardos [1,2,3,*], Matěj Božik [1], Ana Montero [1], Édgar Pérez-Esteve [4], Esther García-Casado [5], Mi-loslav Lhotka [5], Adéla Fraňková [1], María Dolores Marcos [2,3], José Manuel Barat [4], Ramón Mar-tínez-Máñez [2,3] and Pavel Klouček [1,*]

[1] Department of Food Science, Faculty of Agrobiology, Food and Natural Resources, Czech University of Life Sciences Prague, Kamýcká 129, 16500 Praha-Suchdol, Czech Republic; bozik@af.czu.cz (M.B.); anita_mmr@hotmail.com (A.M.); frankovaa@af.czu.cz (A.F.); kloucek@af.czu.cz (P.K.)

[2] Instituto Interuniversitario de Investigación de Reconocimiento Molecular y Desarrollo Tecnológico (IDM), Universitat Politècnica de València, Universitat de València, Camino de Vera s/n, 46022 Valencia, Spain; anberba@upvnet.upv.es (A.B.); mmarcos@qim.upv.es (M.D.M.); rmaez@qim.upv.es (R.M.-M.)

[3] CIBER de Bioingeniería, Biomateriales y Nanomedicina (CIBER-BBN), Av. Monforte de Lemos 3–5, Pabe-llón 11, Planta 0, 28029 Madrid, Spain

[4] Department of Food Technology, Universitat Politècnica de València, Camino de Vera s/n, 46022 Valencia, Spain; edpees@upv.es (É.P.-E.); jmbarat@tal.upv.es (J.M.B.)

[5] Department of Inorganic Technology, Faculty of Chemical Technology, University of Chemistry and Tech-nology Prague, Technická 5, Praha 6, 16628 Prague, Czech Republic; esthergarciacasado@hotmail.es (E.G.-C.); miloslav.lhotka@vscht.cz (M.L.)

* Correspondence: anberba@upvnet.upv.es (A.B.); kloucek@af.czu.cz (P.K.);

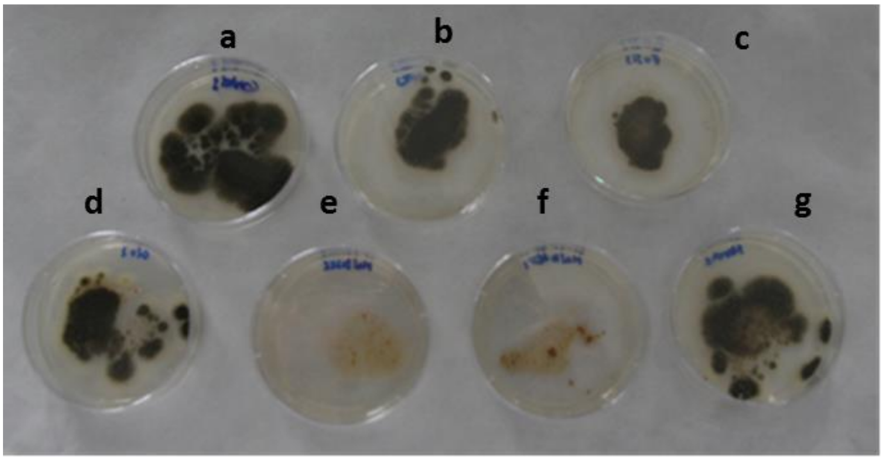

Figure S1. Picture of antifungal activity of control (**a**), pure eugenol (**b**), eugenol encapsulated in silica SMPS-Eu (**c**), eugenol encapsulated in silica and capped with sugar derivative: Glucose-SMPS-Eu (**d**), Maltose-SMPS-Eu (**e**), Maltodextrin-SMPS-Eu (**f**) and Starch-SMPS-Eu (**g**) after 15 days.
